# nf-core/crisprseq: a versatile pipeline for comprehensive analysis of CRISPR gene editing and screening assays

**DOI:** 10.1093/nargab/lqaf214

**Published:** 2026-01-15

**Authors:** Júlia Mir-Pedrol, Laurence Kuhlburger, Marta Sanvicente-García, Metin Yazar, Colm J Ryan, Sabrina Krakau, Gisela Gabernet, Marc Güell, Matteo Bonfanti, nf-core community, Sven Nahnsen

**Affiliations:** Quantitative Biology Center, University of Tübingen, Otfried-Müller-Str. 37, 72076 Tübingen, Baden-Württemberg, Germany; M3 Research Center, Faculty of Medicine, University of Tübingen, Otfried-Müller-Str. 37, 72076 Tübingen, Baden-Württemberg, Germany; Centre for Genomic Regulation, The Barcelona Institute of Science and Technology, Dr. Aiguader 88, Barcelona 08003, Spain; Quantitative Biology Center, University of Tübingen, Otfried-Müller-Str. 37, 72076 Tübingen, Baden-Württemberg, Germany; M3 Research Center, Faculty of Medicine, University of Tübingen, Otfried-Müller-Str. 37, 72076 Tübingen, Baden-Württemberg, Germany; Biomedical Data Science, Department of Computer Science, University of Tübingen, Otfried-Müller-Str. 37, 72076, Tübingen, Baden-Württemberg 72076, Germany; Department of Neurology and Interdisciplinary Neuro-Oncology, University Hospital Tübingen, Hertie Institute for Clinical Brain Research, Eberhard Karls University Tübingen, Otfried-Müller-Str. 27, 72076 Tübingen, Baden-Württemberg, Germany; Department of Medicine and Life Sciences, Universitat Pompeu Fabra, Barcelona, Catalunya 08003, Spain; School of Computer Science, University College Dublin, East Belfield, Dublin 4, Ireland; School of Medicine, University College Dublin, East Belfield, Dublin 4, Ireland; Conway Institute of Biomolecular and Biomedical Research, University College Dublin, Belfield, Dublin 4, Ireland; School of Computer Science, University College Dublin, East Belfield, Dublin 4, Ireland; School of Medicine, University College Dublin, East Belfield, Dublin 4, Ireland; Conway Institute of Biomolecular and Biomedical Research, University College Dublin, Belfield, Dublin 4, Ireland; Quantitative Biology Center, University of Tübingen, Otfried-Müller-Str. 37, 72076 Tübingen, Baden-Württemberg, Germany; M3 Research Center, Faculty of Medicine, University of Tübingen, Otfried-Müller-Str. 37, 72076 Tübingen, Baden-Württemberg, Germany; Quantitative Biology Center, University of Tübingen, Otfried-Müller-Str. 37, 72076 Tübingen, Baden-Württemberg, Germany; Department of Medicine and Life Sciences, Universitat Pompeu Fabra, Barcelona, Catalunya 08003, Spain; ICREA, Institució Catalana de Recerca i Estudis Avançats, Barcelona, Catalunya 08003, Spain; National Facility for Data Handling and Analysis, Human Technopole, 20157 Milano, Italy; Quantitative Biology Center, University of Tübingen, Otfried-Müller-Str. 37, 72076 Tübingen, Baden-Württemberg, Germany; Quantitative Biology Center, University of Tübingen, Otfried-Müller-Str. 37, 72076 Tübingen, Baden-Württemberg, Germany; M3 Research Center, Faculty of Medicine, University of Tübingen, Otfried-Müller-Str. 37, 72076 Tübingen, Baden-Württemberg, Germany; Biomedical Data Science, Department of Computer Science, University of Tübingen, Otfried-Müller-Str. 37, 72076, Tübingen, Baden-Württemberg 72076, Germany; Institute for Bioinformatics and Medical Informatics, University of Tübingen, Otfried-Müller-Str. 37, Tübingen, Baden-Württemberg 72076, Germany

## Abstract

In recent years, CRISPR technology has become widely applied in scientific research, being simpler, cheaper, and more precise than previous gene-editing techniques. This editing technology can be used for various applications, such as gene knockout, gene knock-in, CRISPR activation (CRISPRa), CRISPR interference (CRISPRi), CRISPR screens, base editing, and prime editing. The share of pipelines to analyze the variety of CRISPR editing methods is low, and until now, none of them caters to both gene editing and CRISPR-based functional genomics. Here, we introduce nf-core/crisprseq, a Nextflow DSL2 pipeline for the assessment of CRISPR gene editing and screening assays. The workflow is written in a modularized fashion to allow the easy incorporation of new steps. nf-core/crisprseq is the first generic pipeline enabling the analysis of the broad spectrum of CRISPR designs. We show the performance and usability of the software using publicly available datasets.

## Introduction

In recent years, CRISPR technology has become widely applied in scientific research, being simpler, cheaper, and more precise than previous gene-editing techniques [1, 2]. CRISPR-Cas technology enables precise DNA editing and can be applied to a wide range of research areas, including drug discovery, molecular biology, functional genomics, disease modeling, agriculture, and gene therapies [1, 3]. Its utility extends to understanding gene function, identifying therapeutic targets, and advancing precision medicine [1, 3].

This editing technology can be used for various applications, such as gene knockout (KO) [2, 4], gene knock-in (KI) [4], CRISPR activation (CRISPRa) [5], CRISPR interference (CRISPRi) [6], CRISPR screens [7], base editing (BE) [8], and prime editing (PE) [9].

To manipulate a single gene for a KO, a Cas9 enzyme is guided to the specific gene sequence by a complementary single guide RNA (sgRNA). Cas9 then cuts the DNA at the target site, leading to gene depletion [2]. This approach is widely employed in functional genomics, drug discovery, and disease modeling [3, 7].

In CRISPR KIs, double-strand breaks are repaired by homology-directed repair (HDR) to insert new DNA or entire genes. This technique is used in biotechnology, recombinant protein production, cell line viability, and disease modeling [1].

CRISPRa and CRISPRi enable gene expression regulation for diverse research applications, such as developmental biology and drug resistance screening [3].

More recent techniques include BE and PE, which enable precise single nucleotide substitutions and small insertions and deletions for the latter [8, 9].

Such techniques can be applied to a single gene of interest or used in CRISPR screens. Screening experiments consist of a library of sgRNAs designed to target a wide range of genes simultaneously. Through Next-Generation Sequencing (NGS), the effects of gene depletion or activation on cellular phenotypes can be assessed in a comprehensive manner. This technique facilitates large-scale studies, unraveling genotype–phenotype relationships for drug discovery, as it allows to determine gene essentiality. A gene is considered essential in a cell line if its loss of function leads to the individual’s compromised viability or significant reduction in overall fitness. On a population scale, essential genes are identified by observing intolerance to loss-of-function variants [7].

Analyzing these various experiments involving CRISPR editing requires a collection of tools. Many alternatives for every step of the analysis have been presented in recent years. This complexity challenges the analysis, interpreting the results and extracting meaningful insights from the data. Most importantly, it makes tool benchmarking difficult and, thus, no community agreement for the choice of tools has been established.

Many tools have been developed for the assessment of genome editing. For the analysis of targeted gene editing, these include ampliCan [10], Cas-analyzer [11], CRISPR-Analytics [12], CRISPResso [13], CRISPResso2 [14], CRISPR-GA [15], CRISPRnano [16], CRISPRpic [17], cris.py [18], and CrispRVariants [19]. From these, only CRISPR-Analytics (CRISPR-A) and CRISPResso2 are able to analyze all kinds of CRISPR editions (KO, KI, BE, and PE), and only CRISPR-A is a pipeline built using a workflow manager.

For the analysis of screening experiments, many tools have been developed, such as BAGEL2 [20], CRISPRcleanR [21], MAGeCK [22], Chronos [23], and DrugZ [24], which are often then used in custom in-house scripts, sequentially. Custom in-house scripts, being not open source, limit ease of deployment, optimal use of computational resources, and cluster scalability. However, workflow management systems, such as Nextflow or Snakemake, allow scalable, reproducible, and portable workflows, and simplify application. To this date, only one workflow, MAGeCK-VISPR [25], exists that makes use of a workflow management system, i.e. Snakemake [26] for CRISPR-Cas9 screening analysis. Comprehensive analysis workflows are essential to ensuring the reliability and interpretability of research findings [27], and should achieve several critical objectives to ensure robust and reliable outcomes in scientific investigations. Currently, no analysis workflow allows analysis of both screening and targeted gene editing experiments. The number of CRISPR-mediated research datasets deposited in repositories, such as the European Nucleotide Archive (ENA) [28] or Gene Expression Omnibus [29], and the BioGRID ORCs [30], is continuously increasing.

Here, we introduce *nf-core/crisprseq*, a Nextflow DSL2 (domain-specific language) pipeline for the assessment of CRISPR gene editing and screening assays. DSL2 allows data analysis pipelines to be scaled and modularized. nf-core/crisprseq is the first generic pipeline enabling the analysis of the broad spectrum of CRISPR designs, from targeted gene edits to large-scale functional screens, allowing the characterization of KOs, KIs, base, and PE, and gene modulation experiments. It is the first workflow available that is containerized, improving portability, reproducibility, and easy scalability, thanks to being coded in Nextflow. The workflow is part of the nf-core collection of community-curated, best-practice pipelines. In addition to its broad applicability, we show additional performance in the analysis by contributing new state-of-the-art software and easier usability.

## Materials and methods

### Implementation

nf-core/crisprseq is written in Nextflow DSL2 (domain-specific language), a workflow system for creating scalable, portable, and reproducible workflows [31]. The pipeline is part of the nf-core community, which collects a curated set of analysis pipelines, enforcing best-practice guidelines.

The pipeline provides extensive documentation and community support. Reproducibility is ensured through versioning of each production release and the containerization of all dependency tools. Containerization, seamlessly integrated by Nextflow, is essential for obtaining consistent results and ensuring portability across diverse computing systems. Every pipeline version is tested using a small dataset through continuous integration and a full-size dataset run on AWS.

### Applicability to the analysis of publicly available datasets

In order to assess the versatility of nf-core/crisprseq, we selected public datasets from the ENA, filtered by the keyword “crispr.” We categorized them based on relevant keywords into distinct types of experiments. From 6599 projects, we were able to classify 65.89% into four categories: RNA-seq, single-cell, screening, and targeted experiments. Through this classification, we quantified the percentage of public datasets that our pipeline can effectively analyze.

### Results accuracy benchmarking

Our benchmarking methodology consists of evaluating nf-core/crisprseq’s ability to recover expected indel signal using spike-in data for the targeted subworkflow and to recover a list of essential genes for the screening subworkflow. Both subworkflows were contrasted with their competitive existing tools.

To evaluate the performance of the targeted subworkflow, spike-in samples were used to compare the detected indel rates with the expected rates. Two different datasets were selected. The dataset from Sentmanat, *et al.* (2018) [32] consisted of spike-in samples, which were obtained by mixing a 100% edited sample with a WT sample at proportions 0:1, 2:8, 4:6, 6:4, 8:2, and 1:0, where the edited samples were obtained by KO experiments. The second dataset (PRJEB53901 ENA project) [12] consisted of samples obtained by mixing a 100% edited sample with a WT sample at proportions 1:4 and 1:0, where the edited samples were obtained by site-directed mutagenesis. The nf-core/crisprseq detected indel percentages were compared with the expected percentages. For CRISPR-A, the indel percentage values were obtained from the original publication.

For the evaluation of the screening subworkflow, seven FASTQ sample files from the melanoma A375 cell line screened with the Brunello library using both a modified tracrRNA (trans-activating CRISPR RNA) and a non-modified one were obtained from project PRJNA508200 [33] and processed with both MAGeCK-VISPR and nf-core/crisprseq. Both MAGeCK-VISPR and nf-core/crisprseq were run to compare A375 Brunello replicates against the Brunello plasmid library. In our benchmarking, we defined two design matrices, comparing, first, the dropout screen replicates A and B to the plasmid DNA Brunello, and second, the dropout screen replicates with a modified tracrRNA sequence A, B, and C to the plasmid with a modified tracrRNA sequence. We use the gold-standard gene sets of 1580 essential and 927 non-essential genes to evaluate the gene essentiality metrics provided by the different tools [34].

### Computing performance benchmarking

For the benchmarking of computational resources, the performance of nf-core/crisprseq targeted was benchmarked against CRISPR-A. A workstation was used to run three replicates with both workflows. The resources for all processes were set to 6 CPUs, 32 GB of memory, and 10 h of maximum time for all runs to allow the comparison of the results. To evaluate the resources consumed by a workflow run, a dataset consisting of a 6196 samples was selected from ENA project PRJNA326019 [35] in order to evaluate a real-world experiment.

For running the targeted as well as the screening workflow in a computing cluster with a queuing system, an HPC with the scheduler Slurm with 24 nodes with 32 cores and 64 threads each, with 20 248 GB of RAM was used. The maximum number of tasks allowed to run in parallel by a user was 100. The HPC is shared, and resources per user are allocated by a Fair-share policy.

## Results

### Pipeline overview

nf-core/crisprseq allows the evaluation of the quality of gene editing experiments using targeted NGS data, as well as the discovery of important genes from CRISPR-Cas9 screens using CRISPR pooled NGS DNA data. The two major types of analysis are divided into two subworkflows, named “targeted” and “screening,” respectively.

**Figure 1.**
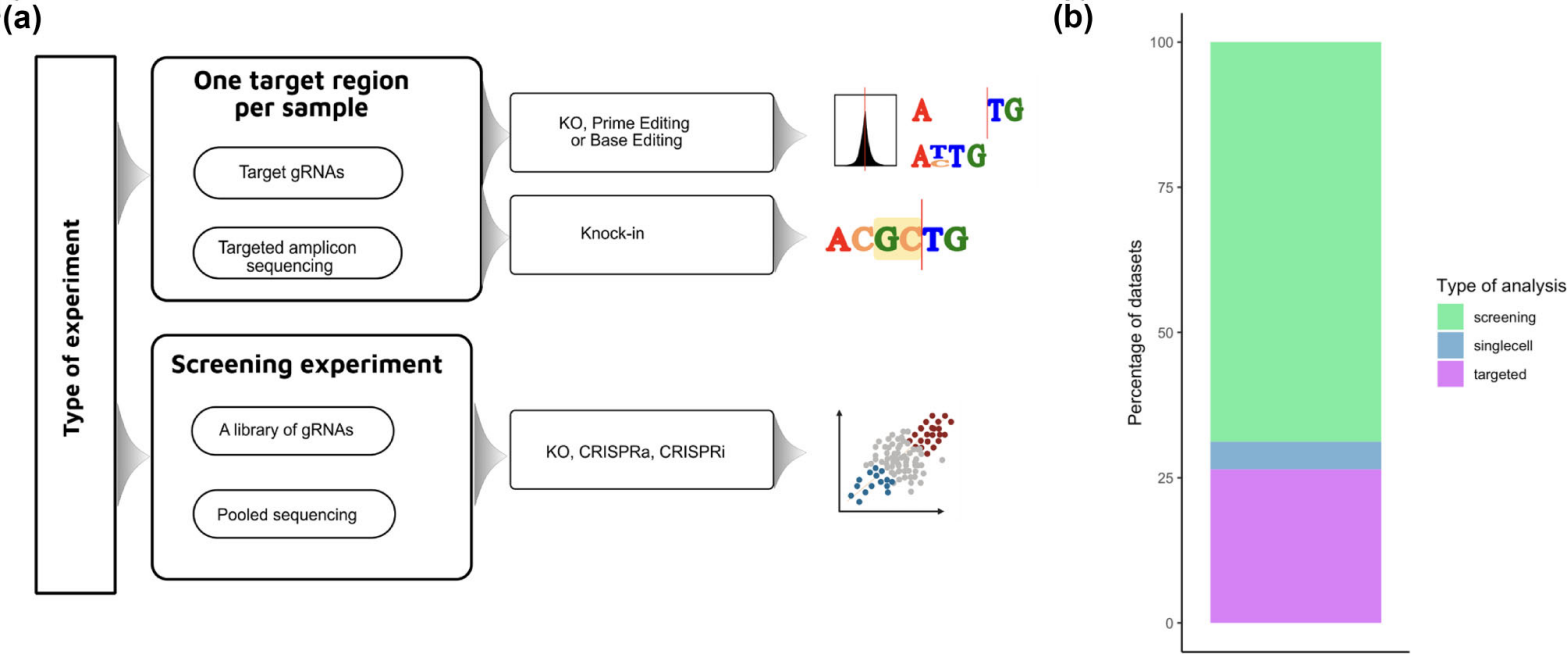
Available analysis types in nf-core/crisprseq. (**a**) Decision schema for each experiment type. Overview of the types of experimental data that can be analyzed with the nf-core/crisprseq pipeline, and the pipeline parameters a user should use to analyze each type of data. (**b**) Analysis type of public datasets. Public datasets containing the keyword “CRISPR” from ENA classified by using keywords according to the type of experiment. Datasets that can be analyzed with nf-core/crisprseq are shown in green for screening experiments, and purple for targeted experiments. Datasets consisting of single-cell data are shown in blue.

The pipeline allows assessing gene KOs, KIs, and base and PE experiments with the analysis of targeted sequencing data, as well as assessing screening experiments, which can be based on KOs, CRISPR activation, or CRISPR interference (Fig. 1a).

The workflow consists of pre-processing steps, including merging of paired-end reads when applicable, quality control of the reads, adapter trimming, and quality filtering.

The targeted subworkflow includes the handling of uni-molecular identifiers (UMIs). Reads are mapped to a reference genome. The assessment of editing outcomes is performed by parsing the CIGAR string. A filtering step is performed to increase the accuracy of the results. The analysis of targeted amplicon reads is based on the existing CRISPR-A tool [12]. Our implementation of the specific targeted workflow is able to improve the overall performance of the analysis in terms of time and resources by increasing the modularization and parallelization of processes (see “Benchmarking” section).

The screening subworkflow includes the alignment of reads to the reference library sgRNAs, followed by statistical analysis of sgRNA abundances. This includes, when applicable, gene-level scoring to ascertain their level of essentiality under the specific experimental conditions. For this, four algorithms are used: MAGeCK RRA, MAGeCK-MLE, BAGEL2, and DrugZ [24]. BAGEL2 requires a list of non-essential and essential genes, which are automatically provided in nf-core/crisprseq for *Homo sapiens* and are the default lists used in the BAGEL2 software. The user can also specify custom lists for other species. Visualizations and pathway analysis are done with FluteMLE [36] for MAGeCK MLE and complementary plots for BAGEL2 and MAGeCK RRA. When the user runs both MAGeCK MLE and BAGEL2 to identify essential genes in their experiments, a Venn diagram is provided showing the intersection with an FDR (false discovery rate) of 10%. This allows to modulate the sensitivity of the analytical approach by allowing varying degrees of inclusivity in identifying essential genes. Considering only the common significant genes between BAGEL2 and MAGeCK MLE effectively reduces the pool of candidate genes to investigate. Users can also identify the significant fitness genes by using the Hitselection module. This module is an implementation of RNAiCut [37, 38] for estimating the best score threshold from ranked gene lists based on the interconnectivity of subgraphs of the BIOGRID physical protein-protein interaction (PPI) network [30]. The core concept of Hitselection is that true positive hits should be densely interconnected on the PPI networks. This module can take as input the gene ranks from MAGeCK, BAGEL2, or DrugZ. This module runs a simulation using a Poisson distribution applied to the ranked list of screened genes. It calculates the -log *P* value to assess the interconnectivity of the actual subnetwork, comparing it to a randomly generated subnetwork where each gene’s degree is matched. The Hitselection algorithm requires for the moment the library to map to genes with a Homo Sapiens EntrezID.

**Figure 2.**
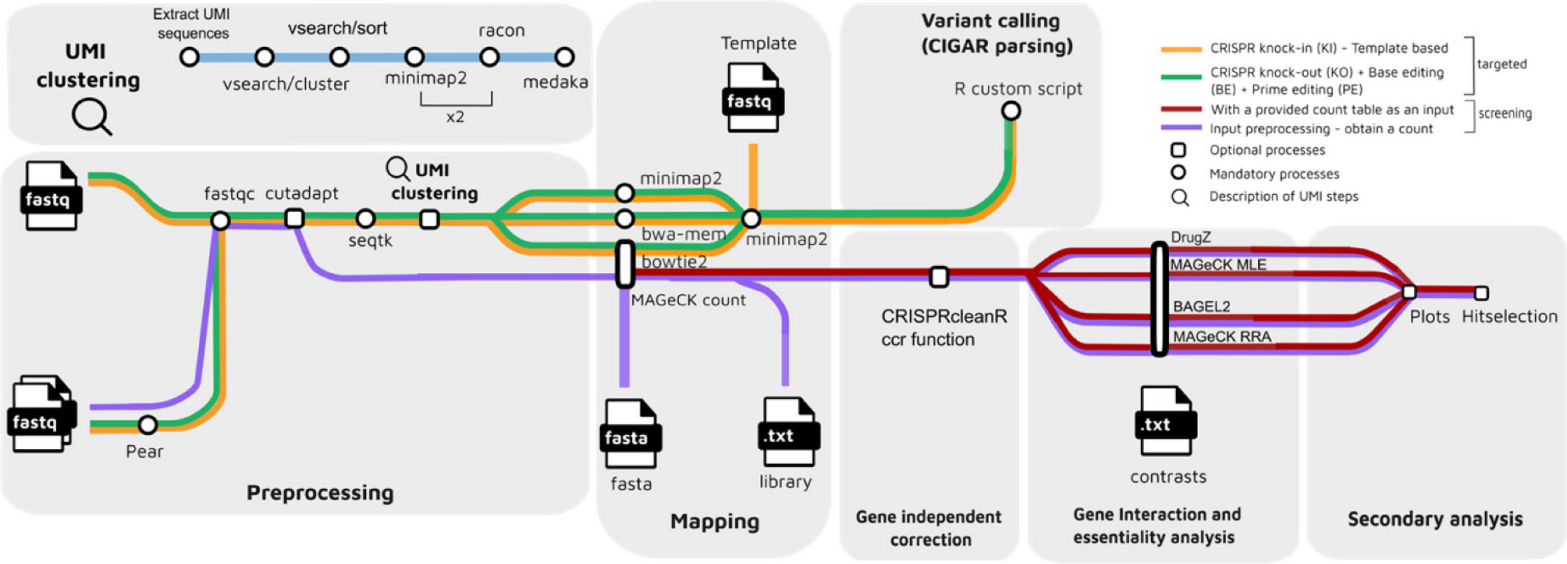
Overview of the analysis workflow. The used tools and the flow of the analysis are represented. The targeted workflow consists of four sections: preprocessing, alignment, optional UMIs clustering, and variant calling. The different analyses of KIs and KOs are shown in orange and green, respectively. The screening workflow consists of four sections: preprocessing, mapping, read normalization, and reporting of gene essentiality. The steps used to analyze the data are shown in purple, an alternative of starting the pipeline providing a count table and skipping the first two sections is shown in red. Mandatory steps are represented with a circle, and optional steps are represented with a square.

An overview of the workflow is provided in Fig. 2.

To assess the versatility of nf-core/crisprseq and its applicability to real-world CRISPR experiments, we classified publicly available datasets. This classification showed that 41.59% of the projects correspond to DNA data, 34% of the projects were unclassified, and 24.3% correspond to RNA data. From the DNA data, nf-core/crisprseq demonstrates its capability to analyze 95.3% of the classified projects, consisting of screening and target experiments (Fig. 1b).

This shows the ability of nf-core/crisprseq to analyze a diverse range of CRISPR-edited datasets and its potential to reanalyze existing datasets in a standardized way.

### Pipeline output and quality control

To ensure transparency and comprehensive quality control, a MultiQC [39] report is provided for both subworkflows. This allows the assessment of the different pipeline steps, including statistics for FastQC and cutadapt steps, and a summary of alignment and indel assessment for the targeting workflow.

To assess the outcome and frequency of targeted edits, a table summarizing read processing statistics and plots illustrating the types of detected editions and indel quality filters are provided. Additionally, the same tables are provided as independent interactive pie charts (Supplementary Fig. S1a and b).

A series of plots is provided to allow a more detailed interpretation of the outcome. These plots include representations of cumulative deletions and insertions based on the read position, and LOGO plots showing the most abundant editions and their percentage in the sample (Supplementary Fig. S1c and d).

The classification of editing outcomes into in-frame and out-of-frame indels, along with reporting their percentages, allows users to quickly assess the success of an experiment by determining whether the expression of a particular gene will be properly disrupted or whether the expected edition is present, and at what frequency.

For the functional screening subworkflow, significant changes in sgRNAs abundance can be determined through different scores depending on the tool used. MAGeCK provides either a negative/positive score through MAGeCK RRA or a beta score through MAGeCK MLE. BAGEL2 provides a Bayes Factor. Each of these scores has its corresponding adjusted *P*-value and distribution plots. For BAGEL2, for instance, the distribution of the Bayes Factor (Supplementary Fig. S2a) and precision-recall curves (Supplementary Fig. S2b) are shown as quality control metrics. In the Hitselection module, the gene rank with the highest -log *P* values serves as a threshold for the list of significant genes in the ordered MAGeCK, BAGEL2, and DrugZ results (Supplementary Fig. S2c).

Quality control plots, enrichment analysis, and distribution plots are provided for MAGeCK-MLE outputs through the FluteMLE module [36]. This allows the visualization of the distribution of the beta values compared to the DepMap data, which contains the dependency scores of 1089 annotated screened cell lines. Exemplary results are provided in the Supplementary Fig. S2. Each subgroup of a gene set from the 9-square plot (Supplementary Fig. S2e) is also available as a table, which is submitted for downstream GO term and Kyoto Encyclopedia of Genes and Genomes (KEGG) [40] pathway enrichment analysis (Supplementary Fig. S2f). nf-core/crisprseq therefore gives the user an overview of up- and down-regulated pathways within the cell line treated in comparison to the DepMap cell lines data.

The full description of the results provided by the pipeline can be found at https://nf-co.re/crisprseq/docs/output.

### Benchmarking

#### Targeted

**Figure 3.**
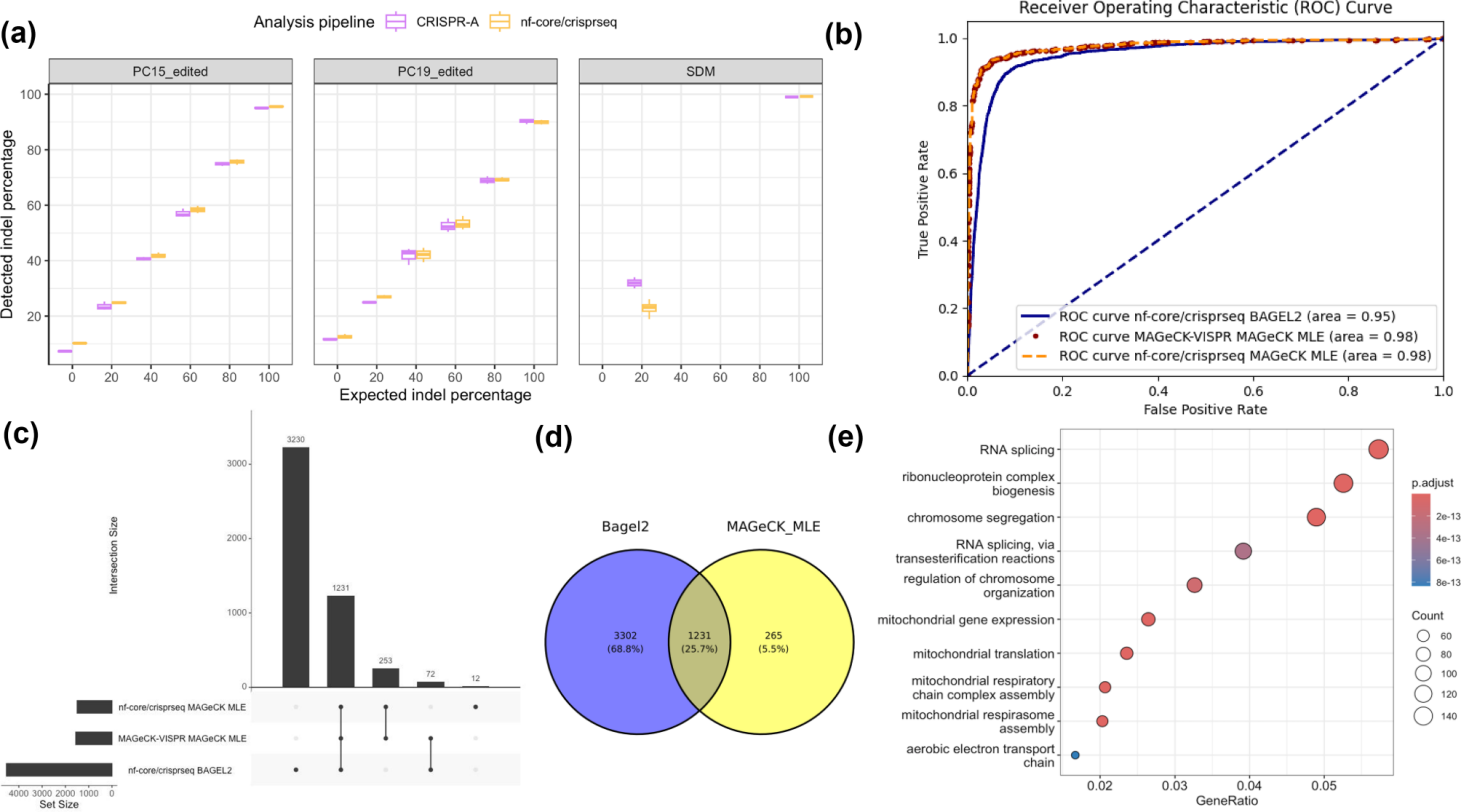
Benchmarking. (**a**) Benchmarking of indels detected with the targeted subworkflow. The detected indel percentage is shown in function of the expected indel percentage. The analyses are performed with nf-core/crisprseq (yellow) and compared against CRISPR-A (purple). The edited samples were obtained by mixing a 100% edited sample with a WT sample at proportions 0:1, 2:8, 4:6, 6:4, 8:2, and 1:0. Two samples, PC15 and PC19, are shown, with three replicates per condition. The side-directed mutagenesis (SDM) samples were obtained by mixing a 100% edited by SDM sample with a WT sample at proportions 1:4 and 1:0. For the diluted samples, 11 replicates were performed with different polymerase chain reaction cycles and total volume. (**b**) ROC curve of the gene essentiality tools in the screening subworkflow. Compares the performance of BAGEL2 from nf-core/crisprseq and MAGeCK MLE from nf-core/crisprseq and MAGeCK-VISPR in distinguishing true positives from true negatives. BAGEL2, trained on a smaller dataset, demonstrates noteworthy effectiveness, but MAGeCK MLE shows slightly superior performance. (**c**) Upset plot of the gene essentiality tools in the screening subworkflow. Shows common intersection gene sets between BAGEL2 from nf-core/crisprseq and MAGeCK MLE from nf-core/crisprseq and MAGeCK-VISPR. (**d**) Venn Diagram of the gene essentiality tools in the screening subworkflow. Comparison between common genes with an FDR < 0.1 from BAGEL2 and MAGeCK MLE. Results were obtained with the nf-core/crisprseq pipeline. (**e**) KEGG Gene Ontology analysis conducted on the 3302 genes identified using BAGEL2 from the screening subworkflow. The analysis highlights several pathways that are integral to cell survival, indicating that these genes play crucial roles in essential cellular functions. Results were obtained with the nf-core/crisprseq pipeline.

nf-core/crisprseq targeted shares a foundation with CRISPR-A; thus, the benchmarking was performed by comparing the detected indel rates and the computational resource usage of both pipelines. CRISPR-A reported comparable results to CRISPResso2, and better performance than other tools such as CRISPRpic, CRISPR-GA, or CrispRvariants [12].

nf-core/crisprseq was used to reanalyze two datasets of samples with a known percentage of indels, and results are reported comparing the detected indel percentage with the expected indel percentage. nf-core/crisprseq shows a comparable ratio of detected versus expected percentage of indels (Fig. 3a).

A dataset consisting of 6196 samples was used to benchmark computational resource usage for the targeted editing workflow. nf-core/crisprseq targeted shares a foundation with CRISPR-A; thus, the benchmarking was performed comparing both pipelines to assess the DSL2 implementation improvement in computational performance. Although the equivalent processes between nf-core/crisprseq and CRISPR-A run with comparable time and memory resource expenses, the overall performance of a complete run favored nf-core/crisprseq. An average run of nf-core/crisprseq on the local workstation takes 14 h 12 m 45 s and uses 222 CPU hours, while an average run of CRISPR-A takes 31 h 59 m 8 s and uses 1919 CPU hours (Supplementary Table S1).

Several factors explain the difference in CPU hours and usage. When using CRISPR-A for the analysis of the full-size dataset, we encountered the need to partition input samples into 12 subgroups to circumvent the run failing, revealing a limitation in its ability to handle a larger volume of samples in a single run. CRISPR-A executed certain processes redundantly, including those associated with UMI clustering or template-based experiments, even when such processes were unnecessary. To provide a more realistic setup, nf-core/crisprseq targeted and screening analyses were performed on a computing cluster with a queuing system (Supplementary Table S2).

#### Screening

For nf-core/crisprseq screening, the average run is 2 h, 31 min, 58 s, and uses 92.65 CPU hours (Supplementary Table S2).

Both MAGeCK-VISPR and nf-core/crisprseq pipelines use FastQC for quality control, and MAGeCK count as a sgRNA counting method. nf-core/crisprseq outputs a MultiQC report for initial quality metrics from FastQC, such as sequence counts, sequence quality for all samples, and also the output of MAGeCK count quality metrics, such as mapping rate, Gini index, and total number of sgRNAs in the library. A general quality control report is not provided for MAGeCK-VISPR, although FastQC is also run, and the output of the MAGeCK count quality metrics is also provided. The output of MAGeCK count outputs the same mapping results for both workflows.

In MAGeCK-VISPR and nf-core/crisprseq, MAGeCK MLE succeeds in identifying common essential genes while not identifying known common non-essentials (Fig. 3b).

In nf-core/crisprseq, BAGEL2, however, finds another 3302 essential genes (Fig. 3d). BAGEL2 is shown to detect subtle changes in overall gene effects, hence explaining the higher number of essential genes detected (Fig. 3c). Unlike null hypothesis-based algorithms like MAGeCK MLE, which need deeper sampling or more pronounced phenotypes, BAGEL2 is optimized for negative selection [41], where genes are identified by their depletion. We performed Gene Ontology enrichment analysis [40] on the 3302 genes detected by BAGEL2, which reveals pathways highly related to cell survival, thereby identifying an additional set of genes of interest (Fig. 3e). The 265 genes detected by MAGeCK MLE only did not lead to any significant pathway enrichment.

The visualization of MAGeCK-VISPR was not possible with the recommended visualization portal, despite thoroughly following the documentation.

## Discussion

Here, we present a novel pipeline allowing researchers to use one framework to analyze a comprehensive collection of CRISPR experiments. nf-core/crisprseq is more user-friendly and has optimized CPU and time usage compared to CRISPR-A. The input preparation for CRISPR-A proved to be intricate, requiring specific file names in a predetermined folder structure and including fake parameters, irrespective of their actual usage. The input for nf-core/crisprseq is facilitated through a sample sheet describing the samples and associated metadata, making rendering the pipeline straightforward and user-friendly. In addition to the pipeline’s applicability, we highlight its broad usability. A typical application of CRISPR-Cas9 technology involves performing a screening experiment to identify relevant targets. These targets are then validated by editing and analyzing them more precisely. Many studies utilize various analysis pipelines to dissect CRISPR-mediated experimental data. This practice introduces complexity, and hinders reproducibility and comparability across studies.

Some examples include the study from Mamedov MR *et al.* [42], where pathways that regulate T cell killing and expression of a particular protein are identified. Three different tools were used to analyze the screening experiments (MAGeCK, GSEA, and Correlation AnalyzeR), and Crispresso2 was used to analyze amplicon sequencing used for validation. In a different study, Schubert OT *et al.* [43] performed base editor screenings to study the effect of individual mutations on protein abundance. They use custom R scripts for the analysis of screenings and Crispresso2 for the characterization of BE efficiency.

In contrast to existing tools and pipelines tailored to a specific type of experiment, our study presents a unique approach to CRISPR data analysis. Using a unified pipeline allows the researcher to perform diverse analyses with greater ease. Our approach simplifies the analysis and enhances reproducibility, making it a valuable resource for the CRISPR research community. The modularity of nf-core/crisprseq also allows for new integrations, whereas MAGeCK-VISPR deploys only tools that relate to the MAGeCK ecosystem, and does not offer easy interfaces for pipeline extensions. Using software such as BAGEL2 or DrugZ is not enabled as part of MAGeCK-VISPR. Undergoing nf-core/crisprseq developments aim at adding functions from the CRISPRcleanR package to correct copy number variation biases [24].

In the era of big data, reusing publicly available datasets holds great potential for accelerating scientific discoveries. Park *et al.* [44], for instance, identified hits from CRISPR screens by correcting the fold change in sgRNA frequency by the actual observed frequency of indel mutations generated by targeted experiments. They successfully applied the sgRNA activity data to previously published screening data to identify novel genes.

The cited studies show the benefit of using publicly accessible datasets in conjunction with the integration of screening and targeted experimental data. This methodological approach helps find genes that may otherwise not be detected.

In a different study, Legut *et al.* [45] combined screening analysis performed with different Cas9 proteins and sgRNAs with different protospacer adjacent motifs with the analysis of indels at the DNA level with amplicon sequencing. They successfully assessed the correlation between protein expression and biases in non-homologous end-joining outcomes. These analyses leverage publicly available datasets to show emerging trends. nf-core/crisprseq would, therefore, allow faster and reproducible reprocessing.

By integrating tools and methodologies for targeted gene editing and large-scale functional screens, nf-core/crisprseq addresses a critical need in the research community for a unified, reproducible, and scalable analysis platform. Our benchmarking demonstrates significant performance, accuracy, and usability improvements compared to existing tools.

nf-core/crisprseq is a reproducible, portable, and scalable workflow for the analysis of CRISPR experiments, enabled by its implementation using the Nextflow workflow management system. Compared to existing tools, it provides improved usability and supports a wide range of CRISPR analysis strategies. Its architecture offers scalability and efficient execution, making it particularly suitable for large-scale applications. Its modular design ensures that it is easily extendable, and facilitates the integration of emerging methodologies and tools as the field evolves.

## Supplementary Material

lqaf214_Supplemental_File

## Data Availability

The code of nf-core/crisprseq workflow is available in [the GitHub repository nf-core/crisprseq, at https://github.com/nf-core/crisprseq] and [Zenodo, at 10.5281/zenodo.7598496], and is shared under the MIT license. Data and scripts used for benchmarking are available in [the GitHub repository qbic-projects/nf-core-crispseq-data, at https://github.com/qbic-projects/nfcore-crisprseq-data] and [Zenodo, at 10.5281/zenodo.15519430] and are shared under the MIT license.

## Appendix

A full list of nf-core community members is available at https://nf-co.re/community.

## Notes

Present address: Sven Nahnsen, M3 Research Center, Faculty of Medicine, University of Tübingen, Otfried-Müller-Str. 37, 72076 Tübingen, Baden-Württemberg, Germany
