# Supplementary material for: nf-core/crisprseq: a versatile pipeline for comprehensive analysis of CRISPR gene editing and screening assays
Source: NAR Genom Bioinform. 2026 Jan 15;8(1):lqaf214. doi: 10.1093/nargab/lqaf214 (PMC12805889; doi:10.1093/nargab/lqaf214)
Supplement: lqaf214_Supplemental_File [file lqaf214_supplemental_file.pdf]

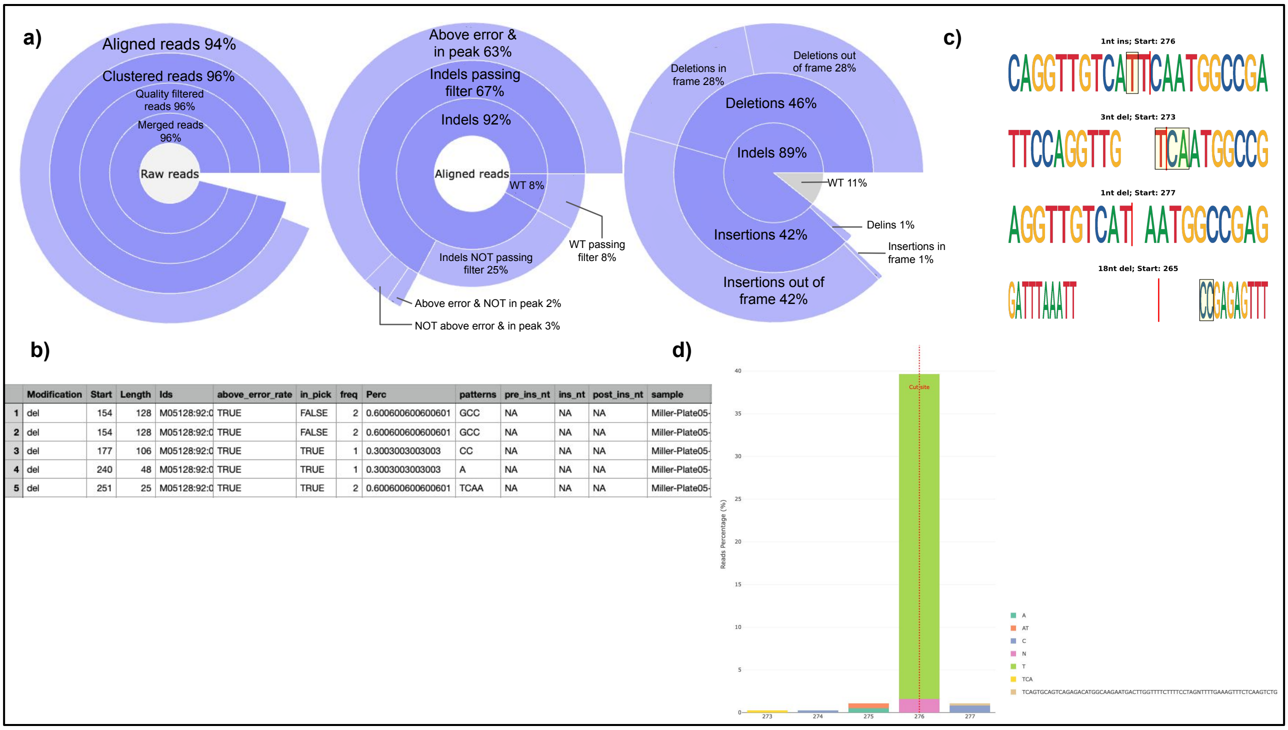

Supplementary Figure 1: Selection of example outputs of the targeted analysis of a 100% edited sample. The shown sample is part of the spike-in dataset from Sentmanat, et. al. (2018). An example of all plots returned by the targeted subworkflow can be found at the nf-co.re/crisprseq website, output section. **a) Pie charts showing the percentage of read classification.** The quality control of reads is represented by the percentage of merged reads, reads passing a quality filtered, reads custard (only relevant for samples using UMIs), and percentage of aligned reads. Aligned reads are then classified in WT and reads with indels, and these are classified as passing QC and error rate filters. Finally, reads that passed the filtering are analyzed to detect the type of indel. **b) Table showing the edition of single reads.** The type and characteristics of the edition from each read is evaluated and reported in a table. Plots are generated from these tables. The depicted table is cropped to show only the relevant columns for space reasons. **c) Logo of the top 4 detected indels. d) Representation of the insertions position.** Insertions are plotted in a bar plot and colored by inserted sequence. The position in the read is represented in axis X, and the cut site is marked in read. The plot shown in the figure is a simplification of the original HTML representation. A similar plot is generated for deletions.

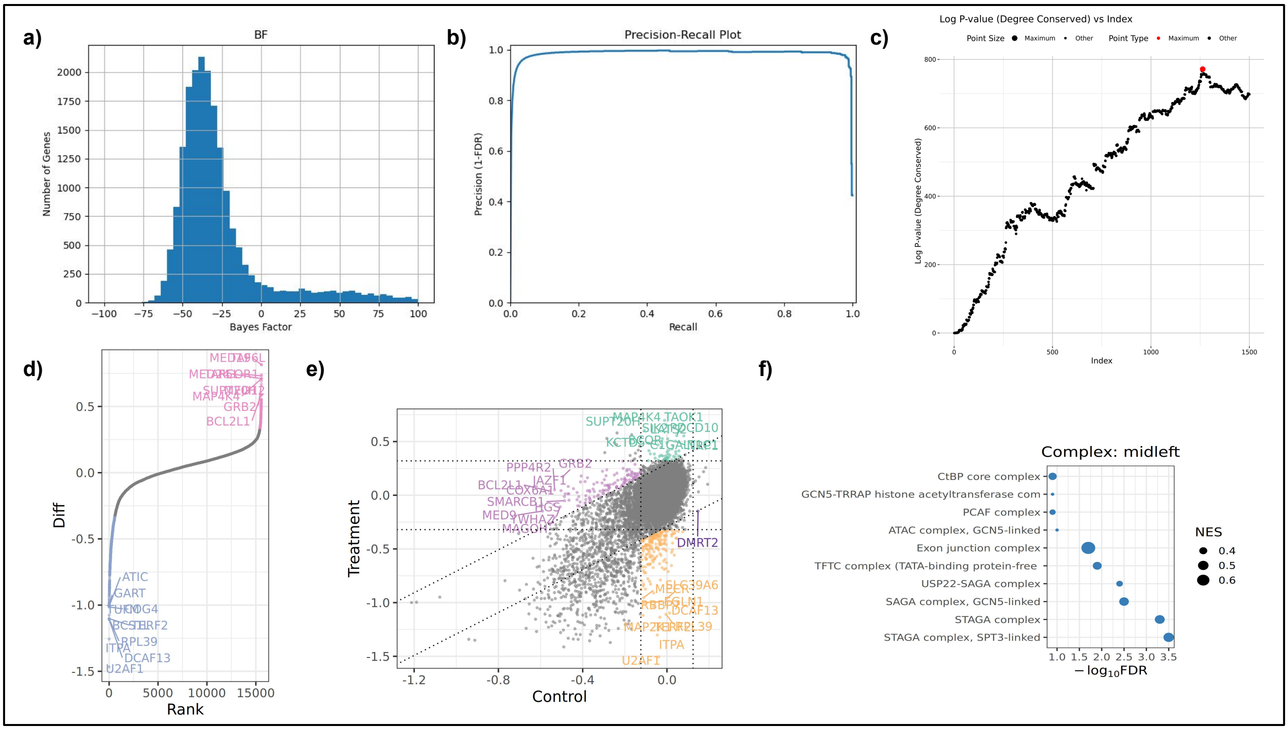

Supplementary Figure 2: Example outputs of the analysis of 1,089 annotated screened cell lines. a) Bayes Factor Distribution as a quality control metric. One expects a high amount of genes with a negative Bayes Factor and a lower distribution with a highly positive Bayes Factor as displayed here. **b) Precision recall curve** output by nf-core/crisprseq screening. A high area under the curve represents both high recall and high precision, where high precision relates to a low false positive rate, and high recall relates to a low false negative rate. **c) Hitselection rank graph for BAGEL2** with the highest -logP values giving the user possible thresholds. **d) Rankview distribution** of MAGeCK MLE output plotted against DepMap data serves as a quality control measure.This shows the genes having the highest difference in beta value between the DepMap cell lines and the Beta values from the comparison defined in nf-core/crisprseq **e) 9-square plot** distribution of Beta values with potential interesting genes. Each quadrant represents the genes which differ from the overall beta values from the DepMap data shown on the x-axis whereas the y-axis shows the beta value of the user's data **f) Gene Ontology** on the mid left quadrant of the 9-square plot. A table of included genes is also provided to the user

nf-core/crisprseq			CRISPR-A						
	time	CPU hours		time run1	time run2	time run3	CPU hours run1	CPU hours run2	CPU hours run3
run1 (6231 samples)	13h 32m 48s	194.50	sub-run1 (537 samples)	2h 51m 4s	2h 52m 36s	2h 52m 20s	28.9	29	29.1
run2 (6231 samples)	15h 54m 34s	236.30	sub-run2 (537 samples)	2h 22m 58s	2h 32m 22s	2h 23m 4s	21.4	22.6	21.4
run3 (6231 samples)	13h 10m 55s	236.10	sub-run3 (537 samples)	2h 18m 11s	2h 51m 52s	2h 18m 7s	19.8	27.2	19.8
			sub-run4 (537 samples)	2h 47m 58s	2h 51m 56s	2h 48m 9s	28.5	29.3	28.5
			sub-run5 (537 samples)	2h 42m 14s	3h 22m 25s	2h 42m 17s	26.8	36.2	26.8
			sub-run6 (537 samples)	2h 39m 21s	3h 32m 14s	2h 38m 40s	25.4	37.8	25.5
			sub-run7 (537 samples)	2h 57m 13s	3h 5m 17s	2h 57m 45s	31.2	32.2	31.3
			sub-run8 (537 samples)	2h 39m 54s	2h 37m 35s	2h 39m 47s	25.3	25.3	25.8
			sub-run9 (537 samples)	2h 14m 11s	2h 31m 34s	2h 32m 56s	18.7	23.3	23.5
			sub-run10 (537 samples)	2h 37m 30s	2h 41m 18s	2h 42m 24s	25.6	25.9	26.6
			sub-run11 (537 samples)	2h 49m 5s	2h 47m 48s	2h 50m 41s	29.1	28.4	29.2
			sub-run12 (324 samples)	2h 0m 56s	2h 1m 43s	1h 42m 2s	19.6	19.6	19.7
average	14h 12m 45s	222	average	31h 59m 8s			1919		

Supplementary Table 1: Run times and CPU hours of a complete run of 6231 samples with nf-core/crisprseq, 3 replicates were run. Run times and CPU hours of 11 subsets of 537 samples and 1 subset of 324 samples with CRISPR-A, 3 replicate was run. The sum of all CRISPR-A sub-runs is used to compute the average. The average of 3 replicates is calculated for both workflows.

	targeted		screening	
	time	CPU	time	CPU
run1	3h 55m 7s	223.70	2h 29m 7s	92.1
run2	4h 7m 39s	230.60	2h 37m 44s	93.4
run3	4h 38m 29s	229.60	2h 27m 14s	91.1
run4	1h 56m 54s	279.70	2h 33m 48s	94
average	3h 39m 30s	240.90	2h 31min 58s	92.65

Supplementary Table 2: Run time and CPU hours of a complete run of 6231 samples with nf-core/crisprseq targeted and 7 samples with nf-core/crisprseq screening, 4 replicates were run for each workflow to minimize the effects of HPC queuing wating times.
